# A Review on the Assessment of Radiation Induced Salivary Gland Damage After Radiotherapy

**DOI:** 10.3389/fonc.2019.01090

**Published:** 2019-10-17

**Authors:** Vincent W. C. Wu, Kit Yee Leung

**Affiliations:** Department of Health Technology and Informatics, Hong Kong Polytechnic University, Kowloon, Hong Kong

**Keywords:** salivary gland, radiation induced damage, radiotherapy, salivary gland recovery, saliva flow

## Abstract

Head and neck cancers are common in Southern China including Hong Kong. Intensity modulated radiotherapy has been the treatment of choice for these patients. Although radiotherapy provides good local control, radiotherapy treatment side-effects are still inevitable due to close proximity of the organs at risk from the target volume. Xerostomia, which is caused due to the damage of salivary glands, is one of the main radiation induced toxicities in post-radiotherapy head and neck patients. This review article discusses the methods for the assessing of radiation induced salivary gland changes including the gland morphology and saliva flow rate. The discussion also includes the recovery of the salivary gland after radiotherapy and how it is affected by the dose. It is expected that the future direction in monitoring the recovery of salivary glands will focus in cellular or molecular levels, and the development of imaging biomarker.

## Introduction

Head and neck cancers are common cancer in Hong Kong that registers about 2,000 new cases annually (1). Owing to the anatomical location and relatively high radiosensitivity of the tumor, radiotherapy is the treatment of choice and satisfactory prognosis has been achieved (2). Currently in Hong Kong, most head and neck cancers are commonly treated by intensity modulated radiotherapy (IMRT), which directs 7–9 beams or rotational beams to the facio-cervical region. The prescribed doses to the primary tumor and cervical lymphatic targets are 72–76 Gy and 54–66 Gy, respectively with the treatment course lasts for over 7 weeks. However, despite the advancement of radiotherapy techniques, radiation induced complications are frequently seen in patients due to the irradiation of the adjacent normal tissues (3–5). The improvement in survival rate implies more patients will experience the late toxicities. Therefore, the protection of organs at risk (OARs) to keep their doses below their respective tolerance become increasingly important.

Xerostomia is one of the common radiation induced complications in head and neck patients after radiotherapy (6–8). The cause of xerostomia is mainly due to the damage of the parotid and submandibular glands, which are the major salivary glands that produce over 80% of saliva (9). The parotid gland is the largest salivary gland located at the preauricular region along the posterior surface of the mandible, while the submandibular gland is the second largest gland located at the submandibular triangle. Since both parotid and submandibular glands are located close to the target volume of head and neck cancers, portions of them are inevitably irradiated to high dose leading to various degree of xerostomia in the patients. Xerostomia causes difficulties in mastication and swallowing and enhances the risks of dental problems; these subsequently degrade the quality of life in the long term survivors (10, 11).

## Radiation Induced Damage to Salivary Glands

In radiotherapy of head and neck cancers, although the detail mechanism of radiation induced xerostomia is not fully known, it is understood that high radiation dose to the salivary glands causes loss and atrophy of acinar cells and granules (12, 13), which leads to morphological changes of the glands and reduces the saliva production. Many studies have reported that there was shrinkage of salivary glands including parotid and submandibular glands after radiotherapy, in which the magnitudes of shrinkage were associated with the total mean doses delivered to these glands (14, 15). Saliva produced from “damaged” parotid and submandibular glands may show reduced levels of the constituents and subsequently affect the digestive and protective processes in the oral cavity. Previous studies have demonstrated that the severity of xerostomia were associated with the hypo-function of the salivary glands due radiation damage, which was found to be dependent on the amount of radiation dose delivered to the glands (16, 17). Because of this, submandibular gland (SMG)-sparing and parotid gland (PG)-sparing techniques have been suggested to minimize the damage of salivary glands in recent studies (18). Several studies including Wang and Eisbruch (19) and Voordeckers et al. (20) supported that the mean parotid dose should be kept below 26 Gy for the preservation of salivary gland function. However, the reduction of dose is not without limit. Kreps et al. (18) reported a mean parotid dose of lower than 20 Gy might increase the risk of local recurrence for head and neck cancers.

## Assessing Xerostomia for Post-Radiotherapy Patients

The onset of radiation induced xerostomia usually starts at the later part of the radiotherapy course in head and neck patients (8). Timely and effective assessments of the xerostomia condition during and after radiotherapy are important for the provision of optimal and timely patient care. Morphology of the glands and saliva flow rate have been studied to assess the post-radiotherapy salivary gland changes and xerostomia condition. They are discussed in the following paragraphs.

### Assessing the Morphology of Salivary Glands

In the 1980's, computed tomography was commonly used to image salivary glands, which was effective to demonstrate duct calculi, diffuse sialectasis, and enlarged lymph nodes (21). To avoid radiation dose to patient, non-invasive imaging modalities such as magnetic resonance imaging (MRI) and ultrasound imaging (UI) have been introduced. These two imaging modalities can visualize changes in terms of gland location, appearance and density as well as the salivary glands function in the case of MRI. Moreover, MRI and UI are prominent modalities in providing detailed image of soft tissue, which is another advantage over CT.

Comparing between MRI and UI for the assessment of post-radiotherapy salivary gland changes, UI is a comparatively affordable solution and has been used in research studies. It is particularly useful in assessing superficial soft tissues. Bialek et al. reported that ultrasonography was useful for detecting acute and chronic inflammation of salivary gland (22). Information, such as size, echogenicity, echogenicity margin sharpness, and echotexture, can be obtained from ultrasound scan for more in-depth analysis of the glands (23). Furthermore, shear wave ultrasound elastography provided easy assessment method of parotid and submandibular gland morphology (24, 25). Regarding the echogenicity of the gland tissue in UI image, it can be classified as hypoechoic, isoechoic, and hyperechoic by comparing with a reference which could be the nearby muscle outside the radiation field. Image echogenicity can also be classified into homogenous for uniform echogenicity and heterogeneous for non-uniform echogenicity. Radiotherapy-induced changes of the submandibular glands, such as reduced gland size, increased heterogeneous with hypoechoic areas and ill-defined margins, are suggested to be associated with the degeneration of acinar cells and loss of parenchymal during and after radiotherapy (26). Furthermore, UI can provide quantitative assessment of the glands using dose-volume histograms generated from scintigraphy (18). In addition, Yang et al. (27) proposed to apply ultrasonic Nakagami distribution, a statistical model for backscatter signals, in the evaluation of gland injury and determining statistical features of the parotid tissues. They also claimed that ultrasonic Nakagami-parameter allowed the segmentation of normal tissue and irradiated tissue. MRI is another non-invasive method of evaluating radiation induced changes (28), but more expensive than UI. MRI can be used to perform quantitative evaluation of early gland changes in post-radiotherapy head and neck cancer patients (29). Kan et al. (30) evaluated the post-radiotherapy parotid gland of head and neck patients using a 1.5 T MRI and concluded that MRI could show internal architecture of the parotid gland. Morphological changes in the irradiated parotid gland were demonstrated by MRI even for low dose irradiation. Nomayr et al. (31) evaluated the appearances of radiation-induced changes in salivary glands using conventional MRI and reported that volume reduction occurred in parotid glands after radiotherapy and hyperintense signal was detected in 22 and 31% of post-radiotherapy parotid and submandibular glands in T2-weighted images, respectively. Volume reduction and increased signal intensity in salivary glands by MRI after radiotherapy were also reported by Wada et al. (32).

Another imaging technique for ductal condition of main salivary gland assessment is sialography which employs the use of contrast injection and radiographic technology. It is useful to detect blockage of the salivary ducts. During the procedure, the patient is instructed to hold the catheter which is placed at the opening of salivary duct, through which the contrast agent is injected, followed by the imaging of the gland by x-ray. Recently, more advanced imaging using MRI has been introduced instead of x-ray to provide 3-dimensional images of the salivary glands in post-radiotherapy patients (33).

### Assessing the Saliva Flow Rate

The flow rate of saliva is an indicator of xerostomia. The accepted range of normal flow for unstimulated saliva and stimulated saliva are above 0.1 and 0.2 mL/min, respectively (34). The calculation of flow rate is performed by dividing the sample volume by the sample collection time (35).

For accurate measurement of flow rate, the method of saliva collection should be reliable and efficient. Before salivary collection, subjects are instructed to refrain from eating, drinking and smoking (17, 36, 37). Mouth rinsing and swallowing is conducted at about 5 min prior to the collection because rinsing the mouth is important to avoid any dilution or food debris that may interfere the result while swallowing helps to hydrate the oral cavity (17). For stimulated salivary collection, stimulation from acid or gum chewing is necessary before salivary collection (19, 38–40). No stimulation is applied for unstimulated collection (38). Saliva collection location is crucial for precise data measurement. Whole saliva is actually a mixed of saliva and oral fluid including oral microbiota which can be collected by spitting or draining (38). For parotid saliva, collection should take place at buccal mucosa. For submandibular saliva, saliva should be collected from floor of mouth near Wharton's duct orifices where the saliva is predominately secreted from submandibular glands together with some from sublingual glands and minor glands (17, 41). Depending on the location and the stimulation type, saliva can be collected by spitting, draining, or suction with the use of Lashley cup or syringe, or by absorbent method with the use of materials such as microsponge, cotton pledget, or synthetic oral swab (38–40). Regardless of the method type, saliva sample should be stored in a sterile container. Collection time depends on the goal of the investigation. In some studies, saliva was collected between 8:00 and 11:00 a.m. to avoid circadian variation in salivary gland function (36, 42, 43). Common collection time is about 5 min and may be longer for unstimulated saliva flow (42).

A more detail assessment of salivary gland function is the “salivary gland scintigraphy,” which employs positron emission tomography (PET) technology (18). Dynamic image acquisition is conducted after intravenous injection of the radioactive tracer 99mTechnetium pertechnetate. Ten milliliters of lemon juice is administrated orally 20 min after the injection to stimulate salivary secretion. Base on the image acquired, time-activity curves are obtained and are used to estimate the maximum salivary secretion (Emax) and minimum salivary secretion (Emin). Salivary excretion fraction (SEF) can then be calculated by the following equation (18):

$$\begin{array}{l} SEF=\frac{\left[\text{Emax }-\text{ Emin}\right]}{\text{Emax}} \end{array}$$

Whereas, the differences in pre-radiotherapy SEF and post-radiotherapy SEF of a patient can be evaluated with SEF ratio (rSEF) using the following equation:

$$\begin{array}{l} rSEF=\frac{\left[\text{preRT SEF }-\text{ postRT SEF}\right]}{\text{preRT SEF}} \end{array}$$

A reduction of more than 25% in rSEF would indicate salivary gland toxicity.

## Recovery of Salivary Gland

Salivary glands have been demonstrated to show recovery after the completion of radiotherapy. Braam et al. (44) and Li et al. (45) studied the parotid gland recovery after radiotherapy in head and neck cancer patients by measuring the stimulated salivary flow rate. They reported that mean dose of <25–30 Gy to the parotid gland could allow complete flow rate recovery. Van Luijk et al. investigated the distribution of stem/progenitor cells in the human parotid gland and suggested that the recovery of parotid glands was depended on the radiation dose and the regenerative capacity of the gland tissue in the irradiated region (46). Seven related longitudinal studies on post-radiotherapy salivary gland recovery using various assessment parameters are discussed below and a summary is given in Table 1.

**Table 1:** Summary of previous studies on the assessing radiation induced changes in salivary gland.

**References**	**Assessing method**	**Subject/control**	**Duration/** **interval**	**Dose** ** (Gy)**	**Outcome**
Murdoch-Kinch et al. (36)	Salivary EGF SF	22	1 year/pre-RT and 3, 6, and 12 months post-RT	21.9	At 6 months post-RT, EGF, parotid SF and total protein increased and returned to pre-RT level at 12 months post-RT in tissues receiving <26 Gy
Almståhl et al. (42)	Buffer capacity Dental caries Oral Microbiota SF Saliva pH	12/12	3 years/6, 12, 24, and 36 months post-RT	51 ± 12^a^ 19 ± 6^b^	Low secretion rate, low buffer capacity, low pH Microorganisms increased Unstimulated secretion improved at 6 months
Chen et al. (47)	Scintigraphy Observer QoL grading	31	2 years/pre-RT and 1, 3, 12, and 24 months post-RT	51.7	For >44.69 Gy, rSEF = 83 ± 32% at 1-year rSEF = 78 ± 22% at 2 year Dry mouth was mostly reported
Gupta et al. (48)	Scintigraphy Observer QoL grading	60	3 years/pre-RT and 3, 12, 4, and 36 months post-RT	50.0^c^ 35.4^d^	Inversed correlation between SEF ratios and mean parotid doses at 3, 12 and 36-months post-RT Salivary toxicity (SEF <45%) Decreased from 3 to 12 months post-RT and became stable afterwards. Threshold for the parotid gland was 35.1, 41.3, 55.9, and 64.3 Gy at 3, 12, 24, and 36 months post-RT, respectively
Strigari et al. (49)	SF Patient-rated XT-related QoL Observer QoL grading	63	2 years/pre-RT and 3, 6, 12, 18, and 24 months post-RT	26–38	XT incidence occurred when the mean dose of the PG, SMG, and TG were >32 Gy Worst XT for patients were shown by TG receiving high mean dose and low irradiated volume
Hey et al. (50)	SF	117	3 years/pre-RT and 1, 6, 12, 24, and 36 months post-RT	34.4^e^ 21.7^f^	The recovery of parotid glands was significant at 2 and 3 years post-RT for parotid mean dose ≤26 Gy
Wang et al. (51)	SF QoL	52	1.5 year/pre-RT and 2, 6, 12, and 18 months post-RT	57.4^g^ 20.4^h^	Correlation was found between RTOG grade and mean dose at 2 and 6 months for SMG XT was less serous for SMG-spared group than non-SMG-spared group at 2 and 6 months post-RT Unstimulated SF for SMG-sparing group was better at all-time points

*SF, saliva flow; Gy, gray; EGF, epidermal growth factor; RT, radiotherapy; SEF, saliva excretion fraction; QoL, quality of life; XT, xerostomia; SMG, submandibular gland; PG, parotid gland; TG, whole organ*.

a *Total dose for patient treated with radiotherapy*.

b *Total dose for patient treated with additional brachytherapy*.

c *Mean dose for ipsilateral parotid*.

d *Mean dose for contralateral parotid*.

e *Mean dose for patients treated with 3D-CRT*.

f *Mean dose for patients treated with IMRT*.

g *Mean dose for patient treated with non-SMG-spared*.

h *Mean dose for patient treated with SMG-spared*.

Chen et al. followed up a group of 31 post-radiotherapy head and neck patients treated by IMRT up to 24 months. Measurements were performed using scintigraphy and observer Quality of life (QoL) grading. They demonstrated an improvement of 5% in rSEF in the second year measurement (78%) compared with that in the first year (83%) (47). Gupta et al. performed a prospective longitudinal assessment of parotid gland function in head and neck cancer patients treated with parotid-sparing radiotherapy. Scintigraphy and rSEF were used to assess the post-radiotherapy changes up to 36 months. The results revealed that there was consistent decline in parotid function even after conformal radiotherapy with moderate recovery over time (48). Still on flow rate measurement, Strigari et al. conducted a prospective longitudinal study on 63 head and neck patients including 44 NPC patients about the duration of xerostomia after radiotherapy. Their results revealed that a reduced salivary flow was observed at 3 months, but after that recovery of flow was observed over time. They also deduced from multivariate analysis that mean gland dose and pretreatment stimulated salivary flow were independent factors for predicting xerostomia (49).

Concerning about the relationship of dose and gland recovery, Hey et al. evaluated the recovery potential of the parotid glands after 3-dimensinal conformal radiotherapy or IMRT on 117 patients. The longest follow up time was 36 months. They found that the gland could reach complete recovery of salivary flow rate if the D_mean_ was <26 Gy at one parotid gland. The volume of irradiation and D_mean_ were indicators of saliva flow and gland recovery (50). Along this line, sparing part of the salivary was found to have effect on gland recovery. Wang et al. researched on the recovery of saliva output and effect of xerostomia grade after IMRT on 52 head and neck cancer patients up to 18 months post-radiotherapy. Applying a contralateral submandibular gland treatment technique could significantly reduce Grad 2-6 xerostomia on patients (51). This group of patients also presented with better mean unstimulated salivary flow rates at each time points and better mean stimulated salivary flow rate at 2 months post-radiotherapy. Based on this, they concluded that recovery of saliva output and grade of xerostomia in post-radiotherapy patients who had contralateral submandibular gland spared were much better those who did not have the submandibular gland spared.

Apart from the dose and volume relationship, some of the bio-factors were also found to be involved in the gland recovery process. Murdoch-Kinch et al. commented that although radiation therapy (RT) causes permanent xerostomia, parotid-sparing radiation therapy (PSRT) ensured recovery of saliva quantity over time. Twelve months after PSRT, parotid glands produced substantial amounts of salivary epidermal growth factor (EGF) and other proteins, eventually approximating pre-radiotherapy levels, with recovery of salivary function (36). In addition, Almståhl et al. conducted a 3-year longitudinal study on the microflora in exosystems and salivary secretion rate after radiotherapy of head and neck patients. They reported that in order to regain a normal, stimulated salivary secretion rate, buffering capacity are the prerequisites to regain an oral flora associated with good oral health (42). Recently, Pringle et al. (52) reported that radiation damaged salivary glands could be restored by the regenerative power of human salivary stem cells, which has the potential in the treatment of xerostomia in future.

## Discussion

Radiation induced xerostomia has been a common complication in radiotherapy of head and neck cancer patients despite of the advancement in radiotherapy techniques. It is logical to expect lower risk of xerostomia if the mean dose to the salivary glands is lowered. Methods to assess the gland condition and xerostomia severity are useful to monitor the progress of the toxicity, where appropriate management can be provided to the affected patients. Our review has discussed the common ways to assess the morphology of the gland mainly using imaging modalities, such as ultrasound, MRI, CT, and scintigraphy using x-ray. Each of them can contribute useful information of the gland condition and their uniqueness in imaging the salivary gland has been identified.

The assessment of the salivary gland function lies mainly on the measurement of saliva flow, which requires a reliable and convenient method of collecting the saliva from patients. The flow volume and flow rate are common indicators of the severity of xerostomia in patients. The details of saliva collection procedure and the parameters for measurement (such as rSEF) have been introduced, which are useful to assess the radiation induced changes in post-radiotherapy patients. In addition, questionnaires for collection patients' subjective feelings on various life aspects related to xerostomia are available. EORTC QLQ-C30 is a questionnaire based on a list of functional measures and symptom measures to assess the QoL of cancer patients. Another cancer-specific questionnaire established by EORTC is the H&N35 which measures QoL based on symptoms (47). Some studies also used patient-rated questionnaire in evaluating salivary gland toxicity or xerostomia severity (49, 53). Patient QoL can be affected by complications associated with salivary dysfunction. Therefore, QoL score in a number of more general dimensions reflects the impact on radiation-induced side effects (54).

Results of several longitudinal studies on salivary gland recovery have been discussed in previous section (Table 1). Apart from them, oral microflora has been reported to reflect the health condition of salivary glands. Saliva pH, saliva buffer capacity and number of microorganism in saliva were lower in post-radiotherapy cancer patients compared with the controls (non-patients). It was speculated that low secretion rate and low buffer capacity were responsible for the proportion of microorganisms in oral cavity and were the cause for the low pH environment (42). Another substance salivary epidermal growth factor (EGF), secreted by the parotid glands, was associated with oral mucosal health. A study has shown that EGF concentration, total EGF, protein concentration, and parotid saliva flow rate were decreased after completion of radiotherapy and restored to the pre-radiotherapy level at 1 year post-radiotherapy (36). However, up to now, the detail mechanism of how salivary gland recover after radiotherapy is still not fully known. This could be a multifactorial event involving dose-volume factor as well as biological factors of the gland and condition of the oral cavity. Therefore, these issues should be investigated in future so as to establish a more comprehensive picture of salivary gland recovery model which can also provide prediction on the degree of recovery in individual patients.

## Conclusion

Assessing saliva gland changes in post-radiotherapy of head and neck cancer patients can be on three main aspects: morphology of the gland, saliva flow rate and saliva content including biomarkers. Imaging modalities play an important part in the monitoring of the morphology of the salivary glands. Ultrasound imaging is advantageous for its convenience whereas MRI is superior in providing details gland textures. In assessing the saliva flow rate, salivary gland scintigraphy can offer more reliable results in which the residual saliva excretion fraction (rSEF) is used. Several studies have proved that salivary recovery took place after completion of radiotherapy. Many of them reported that a greater improvement in salivary function was observed between 1 year and 3 years post-radiotherapy (36, 42, 50). Currently, clinical studies on the recovery of human salivary gland at cellular or molecular level are limited. Leveraging the development in imaging biomarker and studying the recovery of salivary glands at cellular and molecular level will be the future trend (46).

## Author Contributions

KL collected information and drafted the manuscript. VW designed the outline and edited the manuscript. All authors read and approved the final manuscript.

### Conflict of Interest

The authors declare that the research was conducted in the absence of any commercial or financial relationships that could be construed as a potential conflict of interest.

## References

[1] Hospital Authority Hong Kong Leading cancer sites in Hong Kong in 2015 in Hong Kong Cancer Registry (2016).

[2] Wong FC Ng AW Lee VH Lui CM Yuen KK Sze WK. Whole-field simultaneous integrated boost intensity modulated radiotherapy for patients with nasopharyngeal carcinoma. Int J Radiat Oncol Biol Phys. (2010) 76:138–45. 10.1016/j.ijrobp.2009.01.084 19646824

[3] Tsai WL Huang TL Liao KC Chuang HC Lin YT Lee TF. Impact of late toxicities on quality of life for survivors of nasopharyngeal carcinoma. BMC Cancer. (2014) 14:856. 10.1186/1471-2407-14-856 25413127 PMC4247772

[4] Tian YM Guan Y Xiao WW Zeng L Liu S Lu TX. Long-term survival and late complications in intensity-modulated radiotherapy of locally recurrent T1 to T2 nasopharyngeal carcinoma. Head Neck. (2016) 38:225–31. 10.1002/hed.23880 25244494

[5] McDowell LJ Rock K Xu W Chan B Waldron J Lu L. Long-term late toxicity, quality of life, and emotional distress in patients with nasopharyngeal carcinoma treated with intensity modulated radiation therapy. Int J Radiat Oncol Biol Phys. (2018) 102:340–52. 10.1016/j.ijrobp.2018.05.060 30191868

[6] Agulnik M Epstein JB. Nasopharyngeal carcinoma: current management, future directions and dental implications. Oral Oncol. (2008) 44:617–27. 10.1016/j.oraloncology.2007.08.003 18061518

[7] Talmi YP Horowitz Z Bedrin L Wolf M Chaushu G Kronenberg J. Quality of life of nasopharyngeal carcinoma patients. Cancer. (2002) 94:1012–17. 10.1002/cncr.10342 11920470

[8] Mohammadi N Seyyednejhad F Alizadeh Oskoee P Savadi Oskoee S Mofidi N. Evaluation of radiation-induced xerostomia in patients with nasopharyngeal carcinomas. J Dent Res Dent Clin Dent Prospects. (2007) 1:65–70. 10.5681/joddd.2007.011 23277836 PMC3525927

[9] Ortholan C Benezery K Bensadoun RJ. Normal tissue tolerance to external beam radiation therapy: salivary glands. Cancer Radiother. (2010) 14:290–4. 10.1016/j.canrad.2010.03.007 20609609

[10] Jellema AP Slotman BT Doomaert P Leemans CR Langendijk JA. Impact of radiation-induced xerostomia on quality of life after primary radiotherapy among patients with head and neck cancer. Int J Radiat Oncol Biol Phys. (2007) 69:751–60. 10.1016/j.ijrobp.2007.04.021 17560735

[11] Chambers MS Harden AS Kies MS Martin JW. Radiation-induced xerostomia in patients with head and neck cancer: pathogenesis, impact on quality of life, and management. Head Neck. (2004) 26:796–807. 10.1002/hed.20045 15350026

[12] Nagler RM. The enigmatic mechanism of irradiation-induced damage to the major salivary glands. Oral Dis. (2002) 8:141–6. 10.1034/j.1601-0825.2002.02838.x 12108758

[13] Radfar L Sirois DA Structural and functional injury in minipig salivary glands following fractional exposure to 70 Gy of ionizing radiation: an animal model for human radiation induced salivary gland injury. Oral Surg Oral Med Oral Pathol Oral Radiol Endod. (2003) 96:267–74. 10.1016/S107921040300369X 12973282

[14] Wang ZH Yan C Zhang ZY Zhang CP Hu HS Kirwan J. Radiation-induced volume changes in parotid and submandibular glands in patients with head and neck cancer receiving postoperative radiotherapy: a longitudinal study. Laryngoscope. (2009) 119:1966–74. 10.1002/lary.20601 19688858

[15] Teshima K Mukakami R Tomitaka E Nomura T Toya R Hiraki A Radiation-induced parotid gland changes in oral cancer patients: correlation between parotid volume and saliva production. Jpn J Clin Oncol. (2010) 42:42–6. 10.1093/jjco/hyp113 19812062

[16] Ying MTC Cheng SCH Wu VWC Kwong DLW Post-radiotherapy morphological changes of parotid gland are dose- and radiotherapy technique-dependent. Br J Radiol. (2011) 84:1157 10.1259/bjr/30087983

[17] Lou J Huang P Ma C Zheng Y Chen J Liang Y. Parotid gland radiation dose-xerostomia relationships based on actual delivered dose for nasopharyngeal carcinoma. J Appl Clin Med Phys. (2018) 19:251–60. 10.1002/acm2.12327 29664218 PMC5978560

[18] Kreps S Berges O Belin L Zefkili S Petras S Giraud P. Salivary gland-sparing helical tomotherapy for head and neck cancer: preserved salivary function on quantitative salivary gland scintigraphy after tomotherapy. Eur Ann Otorhinolaryngol Head Neck Dis. (2016) 133:257–62. 10.1016/j.anorl.2016.05.003 27291482

[19] Wang X Eisbruch A. IMRT for head and neck cancer: reducing xerostomia and dysphagia. J Radiat Res. (2016) 57(Suppl. 1):i69–75. 10.1093/jrr/rrw047 27538846 PMC4990117

[20] Voordeckers M Farrag A Everaert H Tournel K Storme G Verellen D. Parotid gland sparing with helical tomotherapy in head-and-neck cancer. Int J Radiat Oncol Biol Phys. (2012) 84:443–48. 10.1016/j.ijrobp.2011.11.070 22836056

[21] Bryan RN Miller RH Ferreyro RI Sessions RB. Computed tomography of the major salivary glands. AJR Am J Roentgenol. (1982) 139:547–54. 10.2214/ajr.139.3.547 6981322

[22] Bialek EJ Jakubowsku W Zajkowski P Szopinski KT Osmolski A. US of the major salivary glands: anatomy and spatial relationships, pathologic conditions, and pitfalls. Radiographics. (2006) 26:745–63. 10.1148/rg.263055024 16702452

[23] Cheng SC Ying MT Kwong DL Wu VW Sonographic appearance of parotid glands in patients treated with intensity-modulated radiotherapy or conventional radiotherapy for nasopharyngeal carcinoma. Ultrasound Med Biol. (2011) 37:220–30. 10.1016/j.ultrasmedbio.2010.11.002 21208735

[24] Rahatli FK Turnaoglu H Iyidir OT Kirnap NG Haberal KM Aydin E. Assessment of parotid and submandibular glands with shear wave elastography following radioactive iodine therapy for papillary thyroid carcinoma. J Ultrasound Med. (2019) 38:357–62. 10.1002/jum.14695 30027680

[25] Cindil E Oktar SO Akkan K Sendur HN Mercan R Tufan A. Ultrasound elastography in assessment of salivary glands involvement in primary Sjögren's syndrome. Clin Imaging. (2018) 50:229–34. 10.1016/j.clinimag.2018.04.011 29689477

[26] Cheng SCH Wu VWC Kwong DLW Ying MTC Assessment of post-radiotherapy salivary gland. Br J Radiol. (2011) 84:393–402. 10.1259/bjr/66754762 21511748 PMC3473647

[27] Yang X Tridandapani S Beitler JJ Yu DS Wu N Wang Y. Ultrasonic Nakagami-parameter characterization of parotid-gland injury following head-and-neck radiotherapy: a feasibility study of late toxicity. Med Phys. (2014) 41:022903. 10.1118/1.4862507 24506650 PMC3987707

[28] Ou D Zhang Y He X Gu Y Hu C Ying H. Magnetic resonance sialography for investigating major salivary gland duct system after intensity-modulated radiotherapy of nasopharyngeal carcinoma. Int J Clin Oncol. (2013)18:801–7. 10.1007/s10147-012-0464-y 22892798

[29] Zhou S Qian JJ Xu L Tian Y Fan QH Shen JK. The quantitative evaluation of early radiation-induced changes in the salivary glands using MRI. Zhonghua Yi Xue Za Zhi. (2017) 97:492–5. 10.3760/cma.j.issn.0376-2491.2017.07.004 28260286

[30] Kan T Kodani K Michimoto K Fujii S Ogawa T. Radiation-induced damage to microstructure of parotid gland: evaluation using high-resolution magnetic resonance imaging. Int J Radiat Oncol Biol Phys. (2010) 77:1030–8. 10.1016/j.ijrobp.2009.06.010 19879064

[31] Nomayr A Lell M Sweeney R Lukas P. MRI appearance of radiation-induced changes of normal cervical tissues. Eur Radiol. (2001) 11:1807–17. 10.1007/s003300000728 11511906

[32] Wada A Uchida N Yokokawa M Yoshizako T Kitagaki H. Radiation-induced xerostomia: objective evaluation of salivary gland injury using MR sialography. AJNR Am J Neuroradiol. (2009) 30:53–8. 10.3174/ajnr.A1322 18842755 PMC7051706

[33] Astreinidou E Raaymakers CP Roesink JM Terhaard CH Lagendijk JJ Bartels LW. 3D MR sialography protocol for postradiotherapy follow-up of the salivary duct system. J Magn Reson Imaging. (2006) 24:556–62. 10.1002/jmri.20659 16878305

[34] Humphrey SP Williamson RT. A review of saliva: normal composition, flow, and function. J Prosthet Dent. (2001) 85:162–9. 10.1067/mpr.2001.113778 11208206

[35] Beltzer EK Fortunato CK Guaderrama MM Peckins MK Garramone BM Granger DA. Salivary flow and alpha-amylase: collection technique, duration, and oral fluid type. Physiol Behav. (2010) 101:289–96. 10.1016/j.physbeh.2010.05.016 20515701

[36] Murdoch-Kinch CA Russo N Griffith S Braun T Eisbruch A D'Silva NJ. Recovery of salivary epidermal growth factor in parotid saliva following parotid sparing radiation therapy: a proof-of-principle study. Oral Surg Oral Med Oral Pathol Oral Radiol Endod. (2011) 111:64–70. 10.1016/j.tripleo.2010.09.005 21176822

[37] Pow EH Chen Z Kwong DL Lam OL. Salivary anionic changes after radiotherapy for nasopharyngeal carcinoma: a 1-year prospective study. PLoS ONE. (2016) 11:e0152817. 10.1371/journal.pone.0152817 27031997 PMC4816308

[38] Plemons JM Al-Hashimi I Marek CL. Managing xerostomia and salivary gland hypofunction: executive summary of a report from the American Dental Association Council on Scientific Affairs. J Am Dent Assoc. (2014) 145:867–73. 10.14219/jada.2014.44 25082939

[39] Justino AB Teixeira RR Peixoto LG Jaramillo OLB Espindola FS. Effect of saliva collection methods and oral hygiene on salivary biomarkers. Scand J Clin Lab Invest. (2017) 77:415–22. 10.1080/00365513.2017.1334261 28613965

[40] Vuletic L Peros K Spalj S Rogic D Alajbeg I. Time-related changes in pH, buffering capacity and phosphate and urea concentration of stimulated saliva. Oral Health Prev Dent. (2014) 12:45–53. 10.3290/j.ohpd.a31221 24619782

[41] Dijkema T Terhaard CH Roesink JM Raaijmakers CP van den Keijbus PA Brand HS. MUC5B levels in submandibular gland saliva of patients treated with radiotherapy for head-and-neck cancer: a pilot study. Radiat Oncol. (2012) 7:91. 10.1186/1748-717X-7-91 22704532 PMC3434044

[42] Almståhl A Wikström M Fagerberg-Mohlin B. Microflora in oral ecosystems and salivary secretion rates–a 3-year follow-up after radiation therapy to the head and neck region. Arch Oral Biol. (2015) 60:1187–95. 10.1016/j.archoralbio.2015.04.004 26058004

[43] Ishikawa S Sugimoto M Kitabatake K Tu M Sugano A Yamamori I. Effect of timing of collection of salivary metabolomic biomarkers on oral cancer detection. Amino Acids. (2017) 49:761–70. 10.1007/s00726-017-2378-5 28101653

[44] Braam PM Roesink JM Raaijmakers CP Busschers WB Terhaard CH. Quality of life and salivary output in patients with head-and-neck cancer five years after radiotherapy. Radiat Oncol. (2007) 2:3. 10.1186/1748-717X-2-3 17207274 PMC1779273

[45] Li Y Taylor JM Ten Haken RK Eisbruch A. The impact of dose on parotid salivary recovery in head and neck cancer patients treated with radiation therapy. Int J Radiat Oncol Biol Phys. (2007) 67:660–9. 10.1016/j.ijrobp.2006.09.021 17141973 PMC2001308

[46] van Luijk Pringle S Deasy JO Moiseenko VV Faber H Hovan A Sparing the region of the salivary gland containing stem cells preserves saliva production after radiotherapy for head and neck cancer. Sci Transl Med. (2015) 7:305ra147 10.1126/scitranslmed.aac4441 PMC4964284 26378247

[47] Chen WC Lai CH Lee TF Hung CH Liu KC Tsai MF. Scintigraphic assessment of salivary function after intensity-modulated radiotherapy for head and neck cancer: correlations with parotid dose and quality of life. Oral Oncol. (2012) 49:42–8. 10.1016/j.oraloncology.2012.07.004 22854066

[48] Gupta T Hotwani C Kannan S Master Z Rangarajan V Murthy V Prospective longitudinal assessment of parotid gland function using dynamic quantitative pertechnetate scintigraphy and estimation of dose-response relationship of parotid-sparing radiotherapy in head-neck cancers. Radiat Oncol. (2015) 10:67 10.1186/s13014-015-0371-2 25889705 PMC4373026

[49] Strigari L Benassi M Arcangeli G Bruzzaniti V Giovinazzo G Marucci L A novel dose constraint to reduce xerostomia in head-and-neck cancer patients treated with intensity-modulated radiotherapy. Int J Radiat Oncol Biol Phys. (2010) 77:269–76. 10.1016/j.ijrobp.2009.07.1734 20100642

[50] Hey J Setz J Gerlach R Janich M Hildebrandt G Vordermark D. Parotid gland-recovery after radiotherapy in the head and neck region−36 months follow-up of a prospective clinical study. Radiat Oncol. (2011) 6:125. 10.1186/1748-717X-6-125 21951317 PMC3201902

[51] Wang ZH Yan C Zhang ZY Zhang CP Hu HS Tu WY. Impact of salivary gland dosimetry on post-IMRT recovery of saliva output and xerostomia grade for head-and-neck cancer patients treated with or without contralateral submandibular gland sparing: a longitudinal study. Int J Radiat Oncol Biol Phys. (2011) 81:1479–87. 10.1016/j.ijrobp.2010.07.1990 20934262

[52] Pringle S Maimets M van der Zwaag M Stokman MA van Gosliga D Zwart E. Human salivary gland stem cells functionally restore radiation damaged salivary glands. Stem Cells. (2016) 34:640–52. 10.1002/stem.2278 26887347

[53] Meirovitz A Murdoch-Kinch CA Schipper M Pan C Eisbruch A Grading xerostomia by physicians or by patients after intensity-modulated radiotherapy of head-and-neck cancer. Int J Radiat Oncol Biol Phys. (2006) 66:445–53. 10.1016/j.ijrobp.2006.05.002 16839705

[54] Vergeer MR Doornaert PA Rietveld DH Leemans CR Slotman BJ Langendijk JA. Intensity-modulated radiotherapy reduces radiation-induced morbidity and improves health-related quality of life: results of a nonrandomized prospective study using a standardized follow-up program. Int J Radiat Oncol Biol Phys. (2009) 74:1–8. 10.1016/j.ijrobp.2008.07.059 19111400

